# Individual differences in competent consumer choice: the role of cognitive reflection and numeracy skills

**DOI:** 10.3389/fpsyg.2015.00844

**Published:** 2015-06-17

**Authors:** Michele Graffeo, Luca Polonio, Nicolao Bonini

**Affiliations:** ^1^Department of Information Engineering and Computer Science, University of TrentoTrento, Italy; ^2^Center for Mind/Brain Sciences, University of TrentoTrento, Italy; ^3^Department of Economics and Management, University of TrentoTrento, Italy

**Keywords:** numeracy, CRT, eye movements, decision-making processes, economic choice

## Abstract

In this paper, we investigate whether cognitive reflection and numeracy skills affect the quality of the consumers’ decision-making process in a purchase decision context. In a first (field) experiment, an identical product was on sale in two shops with different initial prices and discounts. One of the two deals was better than the other and the consumers were asked to choose the best one and to describe which arithmetic operations they used to solve the problem; then they were asked to complete the numeracy scale (Lipkus et al., 2001). The choice procedures used by the consumers were classified as “*complete decision approach*” when all the arithmetic operations needed to solve the problem were computed, and as “*partial decision approach*” when only some operations were computed. A mediation model shows that higher numeracy is associated with use of the *complete decision approach*. In turn, this approach is positively associated with the quality of the purchase decision. Given that these findings highlight the importance of the decision processes, in a second (laboratory) experiment we used a supplementary method to study the type of information search used by the participants: eye-tracking. In this experiment the participants were presented with decision problems similar to those used in Experiment 1 and they completed the Lipkus numeracy scale and the Cognitive Reflection Test (CRT; Frederick, 2005). Participants with a high CRT score chose the best deal more frequently, and showed a more profound and detailed information search pattern compared to participants with a low CRT score. Overall, results indicate that higher levels of cognitive reflection and numeracy skills predict the use of a more thorough decision process (measured with two different techniques: retrospective verbal reports and eye movements). In both experiments the decision process is a crucial factor which greatly affects the quality of the purchase decision.

## Introduction

Individual differences in cognitive abilities influence important decisions in many domains. Economic decisions, which strongly affect the well-being of the persons, are a primary example. In this domain, Burks et al. (2009) show that in a large sample of workers (1,000 trainee truckers) those with higher cognitive skills (Numeracy, ability to plan and non-verbal IQ) were more likely to successfully complete their training in a condition where the failure to complete the course involved refunding the training cost. It has also been shown that there is a correlation between higher levels of financial literacy (measured with questions based on the ability to manipulate numbers) and two relevant economic decisions: save for retirement through a private pension plan (Fornero and Monticone, 2011; Lusardi and Mitchell, 2011) and holding stocks (Van Rooij et al., 2011).

A cognitive skill – Numeracy – has a great influence on decisions in the economic context, where often the characteristics of the options are expressed in numerical terms. Data from surveys related to macroeconomic variables that were run in the US, UK, Australia, and Canada indicate that higher Numeracy is associated with higher earnings and higher probability to be employed (Charette and Meng, 1998; McIntosh and Vignoles, 2001; Chiswick et al., 2003; McArdle et al., 2009). At a microeconomic level, Chen and Rao (2007), show how people often mistakenly evaluate the consequences of a sequence of percentage changes and how their intention to purchase a product is affected by these erroneous calculations. Economic decisions can be problematic in particular for lowly numerate consumers, who resort to a variety of coping strategies (e.g., to buy only known brands) in order to overcome the difficulties presented by the quantitative description of goods (Adkins and Ozanne, 2005; Viswanathan et al., 2005). These findings, in addition to highlighting the central influence of individual differences in cognitive skills, suggest that additional research is needed to understand in greater detail how specific cognitive skills affect economic decision-making. The present study focuses on two cognitive skills: Numeracy and Cognitive Reflection. We investigate how different levels in these two cognitive skills affect the decision process and the quality of choice. The role of Numeracy is investigated in Experiment 1. The role of Numeracy and Cognitive Reflection are jointly investigated in Experiment 2. The quality of choice is investigated by using scenarios where the participants choose one of two alternative offers of the same product, with one option that is more valuable than the other. The use of problems that have an optimal solution allows us to investigate the role of cognitive skills on the quality of choice, rather than focusing on choice consistency as in the more familiar literature on framing, inter-temporal choices and similar decision tasks. For a discussion on the differences between problems that involve optimal choices or consistent choices see Reyna and Brainerd (2008).

### Numeracy

“Numeracy” has been defined as the ability to comprehend, use and attach meaning to numbers (Peters et al., 2006; Reyna and Brainerd, 2008; Reyna et al., 2009). In particular, Numeracy includes the ability to compare magnitudes, and comprehend ratio concepts (including fractions, proportions, percentages, and probabilities). Several studies tried to determine which are the psychological processes underlying the ability to correctly comprehend and manipulate numeric information. Different viewpoints have been suggested: for example Lipkus and Peters (2009) propose a model based on a dual system approach (see Sloman, 1996; Stanovich and West, 2000; Kahneman and Frederick, 2002; Kahneman, 2003) where the two systems are responsible for different steps of the comprehension and manipulation of numbers. System 1 is activated during the initial step of this process: when people perceive a numeric stimulus they intuitively evaluate its magnitude (Peters et al., 2008). A subsequent step – number manipulation – is based on logic operations which are part of System 2. Finally, these operations (generated by Systems 1 and 2) concur to the formation of a meaning/interpretation of the numeric information. In this model the functions of the two systems are strictly intertwined and this is coherent with the idea that good choices are most likely to emerge when the two modes work in concert (Damasio, 1994). Fuzzy-trace theory (see Reyna and Brainerd, 1995; Reyna, 2004) also proposes a dual system approach, which includes verbatim processing (based on precise, detailed, or quantitative representations) and gist processing (based on fuzzy, gist, or qualitative representations). According to this theory some biases (e.g., ratio bias, denominator neglect) result from gist-based processes: people focus their attention on what they believe to be the more salient piece of information (e.g., the numerator in a fraction or a probability estimate) at the expense of a more complete description of a problem. However, the fuzzy-trace theory considers the gist-based processes as more advanced, compared to the verbatim processes, because they focus on the essential elements of a problems (see Reyna and Brainerd, 1995; Reyna and Lloyd, 2006). Finally, the frequency hypothesis (Gigerenzer, 1994; Gigerenzer and Hoffrage, 1995) posits that humans have directly experienced frequencies throughout their evolutionary history, making frequencies easier to understand compared to decimals or probabilities, which are expressed on a normalized scale that does not occur in nature. However, Chapman and Liu (2009) report an experiment where the benefits of the natural frequency format occurred primarily for participants who were high in Numeracy. This result suggests that in the general population the correct comprehension of arithmetic problems can not be assured only by the use of the natural frequencies. For a comparison of the three approaches mentioned above see Reyna and Brainerd (2008). Del Missier et al. (2012) describe Numeracy as a general cognitive ability, which has a sort of intermediate level between basic abilities (the executive function, i.e., control processes involved in the regulation of cognition, see Rabbitt, 1997; Miyake et al., 2000; Royall et al., 2002; Salthouse, 2005) and more complex skills (e.g., the capacity to correctly apply a series of decision rules that involve computations). In particular, a more developed supervision ability – which includes the monitoring and revision of working memory contents and the capacity to appropriately inhibit irrelevant information and responses – is associated with higher Numeracy scores (see also Mäntylä et al., 2007 for a description of this executive function).

### Numeracy and Choices

The effect of Numeracy on the evaluations and choices has been investigated in different fields such as risk perception, risk communication, pro-social and health-related decisions (see: Lipkus et al., 2001; Hibbard et al., 2007; Peters et al., 2007a,b, 2011; Nelson et al., 2008; Greene and Peters, 2009; Lipkus and Peters, 2009; Dickert et al., 2011). The findings of these studies show, for example, that the economic decisions of low numerate people (e.g., the willingness to donate for charity) are more influenced by changes in numeric presentation format than those of people with higher numeracy (Dickert et al., 2011). Greene and Peters (2009) found that some contracts, heavily based on numeric information (e.g., health insurance plans) are difficult to understand especially for low numerate people. Consequentially, Peters et al. (2007a) found that strategies which reduce the cognitive load are particularly helpful for low numeracy consumers. Cokely and Kelley (2009) show that there is a relationship between cognitive abilities (Numeracy and Reflectiveness, measured through the Cognitive Reflection Test (CRT), decision processes and number of choice that maximize the expected value in a series of gambles. Overall, these studies suggest that highly numerate people draw a more precise and coherent mental representation of numeric information, compared to low numerate people. In addition, highly numerate individuals are more skilled in the integration of numeric information and their choices are less influenced by the way the numeric information is framed, as if they can create a single mental representation of the numeric information, common to different presentation formats. On the contrary, low numerate people tend to focus their attention on some pieces of information, without an adequate integration process (Slovic et al., 2000; Peters et al., 2006, 2008).

## Experiment 1

The goal of Experiment 1 is to study the relationship between Numeracy and the decision process used to choose between different offers of the same product, and how the decision approach affects the quality of choice. Imagine a consumer who considers two alternative deals on the same product. Shop A offers a mobile phone at 180aaax020ACaaa with a 15% discount. Shop B offers the same mobile phone at 220€ with a 25% discount. There are no transaction costs (e.g., the two shops are at the same distance from home).

From the Numeracy literature reported in the Section “Introduction,” we expect that highly numerate people make a computational effort to select the best deal. For example, in order to find the cheapest option, they calculate the final price of both deals. In more general terms they use a specific decision process that includes performing all the arithmetic operations needed to have a complete description of all the alternatives. We refer to this decision process as a “complete decision approach” to the purchase decision task. We expect that the use of a complete approach to the purchase decision task is positively associated with the selection of the best deal.

On the contrary, we expect that it is less likely that consumers with lower Numeracy scores compute the final prices of the two deals. This might be due to several factors. For example, the math computation is particularly tiring for people with low Numeracy or they do not trust their numerical skills. Regardless of the actual determinant, this also implies a specific decision process in which consumers do not try to perform all arithmetic operations needed to calculate the two final prices (e.g., they compute only one final price and then give up). Alternatively, they simply do not perform any computation at all (e.g., they compare the initial prices or percentage discounts). We refer to this decision process as an “incomplete decision approach” to the purchase decision task. We expect that the use of an incomplete decision approach is positively associated with the selection of the worst deal.

### Method

One-hundred and fifty-three consumers participated in this study and 42% of the participants were males. In order to increase the ecological validity of our study, we collected the data at the exit of a shopping mall and asked the participants to choose one of two alternative offers. Three decision scenarios were presented with E-Prime 1.0 (Psychology Software Tools, Inc) software (Schneider et al., 2002a,b), and the participants were randomly assigned to one of the three scenarios, creating three groups of equal size. The logic behind our choice of presenting more than one scenario is that the preferences of the participants could be affected by specific pieces of numeric information, in a way which was difficult to predict^1^. We believed that presenting more than one scenario could reduce the risk that our findings were overly influenced by the specific characteristics of a scenario. Each scenario uses a different product (Mobile phone, DVD player, Microwave oven), and the findings show that the choices are not affected by the kind of product. For this reason, in the next section the analyses do not include the factor “product^2^.” Each scenario describes two shops that offer a deal on the same product: one shop offers a low percentage discount on a low initial price (best deal), while the other shop offers a high percentage discount on a high initial price (worst deal); note that the final prices were not shown to the participants. It may be possible that people associate higher prices with better quality but since we specified that exactly the same product was sold in the two shops it is unlikely that this association occurred during the experiment. The participants decided which deal they preferred (we reversed the order of presentation of the two offers for half of the participants, but there is no order effect). **Table 1** reports prices, discounts, and final prices.

**Table 1:** List of the products for shop A and B, with the corresponding initial prices and discounts, Experiment 1.

	Shop A			Shop B		
Product	Initial price (€)	Discount (%)	Final price (€)	Initial price (€)	Discount (%)	Final price (€)
Mobile phone	180	15	153	220	25	165
DVD player	140	30	98	180	40	108
Microwave oven	70	20	56	90	30	63

To investigate the decision process underlying the consumer choice we asked for retrospective verbal reports, a technique widely used in the cognitive science (Ericsson and Simon, 1980) and in the J/DM literature as well (for the use of similar procedures, see Payne, 1976; Rettinger and Hastie, 2003; Cokely and Kelley, 2009). The rationale for the use of retrospective verbal protocols in our study is that when people consciously and deliberately consider information (e.g., the computation of the absolute value; the subtraction of the absolute value from the initial price), these processes should be observable in participants’ protocols (Sloman, 1996; Evans, 2008). First we reminded the consumers of the numerical values of the deals (e.g. “Shop A offers a 25% discount on a mobile phone that costs 220€”), then we asked “When you decided in which shop to buy, did you calculate the corresponding final price (i.e., 220€ minus 25%)?” (Yes vs. No). The question was repeated for the second deal on the same product.

The verbalization task allows us to measure how many consumers computed zero, one, or two final prices before making the choice. In order to choose the best deal the consumers should create a complete description of all the possible alternatives, which in this scenario means to calculate two final prices (complete decision approach). Consumers who calculated one or zero final prices had a partial/incomplete description of the options and their choices were most likely based on an approximate estimation of the final prices (partial decision approach).

Finally, the consumers completed the Lipkus et al. (2001) questionnaire. This questionnaire is commonly used in the literature to measure people’s Numeracy skill as discussed in the Introduction. In the following analyses we use the number of correct answers as a Numeracy measure. It is important to note that many items of the Lipkus Numeracy scale ask participants to transform numeric information from the percentage format to the frequency format or vice-versa. The solution of these questions require operations that strongly resemble the operations needed to calculate the absolute value of a discount and the corresponding discounted price, i.e., the operations needed to find the best purchase decision. For this reason we believe that the Lipkus scale is particularly appropriate for measuring the arithmetic skills used in our experiment.

### Results and Discussion

Our first analysis studies the correlation between the number of correct answers given to the Numeracy test (the scale ranges from 0 to 11, the mean score is 7.39, SD: 2.3, median: 8) and the time needed to complete the test; the result is not significant [*r*(153) = -0.12, *p* = 0.14]. This result indicates that low Numeracy consumers did not try to finish a potentially distressing task as fast as possible, so it is unlikely that the low Numeracy score is caused by a lack of effort or motivation.

**FIGURE 1:**
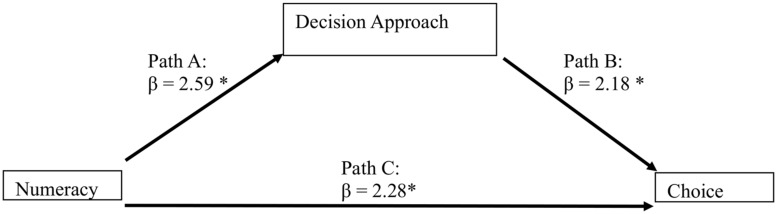
**The mediation model for Experiment 1 shows that Numeracy predicts the Decision Approach, which in turn predicts choice.** ^∗^*p* < 0.05.

In order to analyze the relationships between Numeracy (continuous I.V.), decision approach (binary Mediator; complete vs. partial decision approach) and choice (binary D.V.; best vs. worst deal), we used a mediation model following the regression approach proposed by Baron and Kenny (1986). Given that the dependent variable is dichotomous, we used a series of binary logistic regressions, as described in Preacher and Hayes (2004, 2008) and Hayes (2009, 2012) and the resulting beta coefficients indicate the log odds of the probability of selecting the best option. The mediation model hypothesizes an effect of the independent variable on the dependent variable through a third explanatory variable, the mediator. The independent variable affects the mediator variable, which in turn affects the dependent variable. In this way the mediator clarifies the nature of the relationship between the independent and dependent variables (MacKinnon, 2008). The mediation model is described in **Figure 1**; **Table 2** we report the descriptive statistics of the independent and dependent variables.

**Table 2:** Descriptive statistics of the independent and dependent variables of Experiment 1.

	Average	SD


Numeracy – number of correct responses	7.39	2.3
Time needed to complete the decision task (ms)	55025	26270

	**Proportions**	

Decision approach	Complete approach: 55.6%	Partial approach: 44.4%
Choice	Best option: 83.7%	Worst option: 16.3%

Path C: consumers with low Numeracy chose the worst deal (higher final price) more frequently than the highly numerate consumers [Wald(1) = 5.25; β = 2.28; *p* = 0.022]^3^.

Path A: consumers with low Numeracy used the partial decision approach more often than the highly numerate consumers [Wald(1) = 6.76; β = 2.59; *p* = 0.009].

Path B: indicates a mediation effect: when we include the variable “decision approach,” which has a significant effect on choice [Wald(1) = 4.74; β = 2.18; *p* = 0.030], Numeracy does not have a significant effect [Wald(1) = 3.57; β = 1.90; *p* = 0.059]. We measured the indirect effect of the model (the amount of mediation, i.e., the reduction of the effect of the initial independent variable on the outcome) through a single bootstrapping test (see MacKinnon et al., 2002; Shrout and Bolger, 2002; Preacher and Hayes, 2004, 2008). The result confirms the mediation effect [indirect effect: 95% CI (0.002, 0.138), based on 5000 bootstrap samples]. This result is coherent with our prediction.

The analyses show that consumers with high Numeracy scores chose the best commercial offer more frequently. At the same time, they were more likely to choose the offer after having calculated both final prices, that is by the means of a complete decision approach to the purchase decision task. On the contrary, consumers with low Numeracy often chose by means of a partial decision approach. They reported that they made the choice without doing any calculation at all, or by the computation of just one final price. This indicates that they chose without knowing the real economic values of the alternatives (the partial decision approach has been used 58.8% of the times by consumers with numeracy scores equal or lower than the median and 41.2% of the times by consumers with numeracy scores higher than the median).

The next step of the mediation analysis shows that consumers who used a complete decision approach chose the best discount more frequently, compared to the consumers who used a partial decision approach [90.6% vs. 75%, χ^2^ (1) = 6.7; *p* = 0.01]. The final step of the mediation model indicates that the decision approach is a more relevant predictor of purchase decision skill than Numeracy. In other words, consumers with higher Numeracy scores are more likely to use the complete decision approach than consumers with low Numeracy, but if they use the partial decision approach their choices are not different from the choices of the consumers with low Numeracy. In fact, if we consider only the consumers who used a partial approach, a logistic regression analysis indicates that Numeracy does not predict choice quality [Wald(1) = 0.01; β = -0.1; *p* = 0.92]. On the contrary, if we consider only the consumers who used a complete approach, Numeracy significantly predicts choice quality [Wald(1) = 7.40; β = 2.72; *p* = 0.007]. As a final analysis, we compared how much time the consumers needed to decide with the partial or the complete approach and a *t*-test indicates that consumers who use the partial approach are significantly faster than those who use the complete approach [*M* = 48.5 s vs. *M* = 60.2 s; *t*-test (151) = -2.8; *p* = 0.006]. A similar result has been shown by Cokely and Kelley (2009).

### Conclusion

The mediation model shows that the decision approach is a critical factor of the decision process: the partial approach reduces the ability to find the best option, even for the highly numerate consumers, while when the consumers use the complete approach the likelihood of selecting the best option depends on Numeracy. The mediation model shows that some highly numerate consumers use a partial decision approach to solve the economic decision problem, and this cannot be due to a lack of numeric competence. Said differently, the use of a partial decision approach to solve the economic problem might depend on plural factors, rather than just Numeracy. To our view, one relevant factor is the attitude about how to approach a decision problem and we used the CRT to explore a specific element of this general attitude: the propensity to act reflexively when facing a decision problem. The role of Cognitive Reflection is fully investigated in the next experiment as a potential factor, and compared to Numeracy as a predictor of the quality of the purchase decision.

### Reflexiveness and the Cognitive Reflection Test

Reflectiveness is a broad psychological notion. It has been extensively studied in different fields of psychology (e.g., cognitive, clinical, social, and consumer psychology) and economics (e.g., judgment and decision-making). While researchers in psychology have focused more on the ability to control impulsive behavior (Eysenck and Eysenck, 1978; Rook and Fisher, 1995; Puri, 1996; see also Grecucci et al., 2014 on the role of impulsivity in compulsive gambling), economists and decision researchers were more focused in hyperbolic discounting, or more generally in the ability to delay gratifications (Monterosso and Ainslie, 1999). Studies in psychology have emphasized the distinction between cognitive processes that occur spontaneously and require a moderate level of attention and processes requiring effort, motivation, concentration, and the execution of complex learned rules. According to Stanovich and West (2000) and Evans (2008) these different cognitive processes would be supported by two different systems: a basic system (System 1) of effortless and rapid perception that drive intuitive and elementary operations and a more sophisticated system (System 2) which is slow, analytical, and rational.

Following this theoretical framework, the CRT was conceived as a measure of the ability to control a high accessible (incorrect) answer in favor of a less accessible (correct) answer which requires further deliberation. In the words of the author “CRT measures ‘Cognitive Reflection’ – the ability or disposition to resist reporting the response that first comes to mind” (Frederick, 2005, p. 35). Many researchers since the original publication have used and still use the CRT as a measure of Cognitive Reflection (see for example, Cokely and Kelley, 2009; Fernbach et al., 2013). In particular, we use the term “cognitive impulsivity” to describe the cognitive style of people with low CRT scores.

The CRT consists of three mathematical word problems that elicit spontaneous, intuitive and incorrect answers. For example, in one of the CRT problems participants face the following question: “A bat and a ball cost $1.10. The bat costs $1.00 more than the ball. How much does the ball cost?” The intuitive answer to this problem is “10 cents,” whereas the correct one is “5 cents.” Frederick’s study shows that CRT is associated to the performance in several decision tasks. For example, in the inter-temporal decision task it has been found that participants with high CRT are more patient, e.g., they chose more frequently the larger reward at a later time, instead of a smaller immediate reward (e.g., they prefer 140$ in a year to 100$ now). In risk choice tasks, it has been found that high CRT participants are more willing to take a gamble that has a high expected payoff (instead of a sure gain), compared to low CRT participants (e.g., they choose a 90% to gain 500$ over a sure gain of 100$). Subsequent studies report results which are in line with Frederick’s findings: for example, Oechssler et al. (2009) show that people with high scores in the CRT are much less subject to the conjunction fallacy in Linda’s problem and they update probability estimates more correctly, compared to low CRT people. According to Campitelli and Labollita (2010), high CRT people are better (compared to low CRT people) at using their background knowledge in order to guess the right answer to question on topics about which they know little of. It has been shown that there is a relationship between the ability to answer correctly to the CRT and Numeracy questions. Frederick (2005) reports that there is a moderate correlation between CRT and the SAT Mathematics scores (0.46). Del Missier et al. (2012) showed that the CRT scores depends on the executive functions of Monitoring and Inhibition (the capacity to appropriately inhibit irrelevant information and responses and the capacity to monitor and revise working memory contents). However, this effect is partially mediated by Numeracy (measured with the scale by Lipkus et al., 2001). Overall, the experimental findings indicate that the CRT scores depends on (at least) two elements: reflexiveness and numeracy.

Nofsinger and Varma (2007) found results similar to those of Frederick interviewing a sample of financial advisors. In the domain of the economic decisions Moritz et al. (2013) show that managers with high CRT scores take more profitable decisions. Mixed evidence has been found about the relation between CRT scores and the answers given to moral dilemmas (Paxton et al., 2012; Royzman et al., 2014).

## Experiment 2

In Experiment 2 we investigate the role of reflectiveness on the decision process and the quality of the purchase decision. In Experiment 1, we found that consumers who use a partial approach make their decisions faster and choose the best offer less often compared to those who use a complete approach. This decision pattern is associated to a low level of numerical skills. However, reported findings suggest that, in addition to Numeracy, cognitive impulsivity might inhibit people from making a full computation of final prices. For example, cognitively impulsive consumers might well focus on accessible information to guess which is the best option (e.g., the product with the lowest initial price), although they have the cognitive resources to make the extensive computation (e.g., high Numeracy). Based on previously presented findings, CRT seems to us a good tool to measure cognitive impulsivity in a commercial context. For example, low CRT consumers might rely on accessible information (e.g., lowest initial price; higher percentage discount) to quickly select what looks to them the best deal. This might be the first thought that comes to mind to an impulsive consumer. If not corrected, it might lead the consumer to select the suboptimal offer. This impulsive consumer choice should be reflected also in the decision process. For example, we also expect that low CRT consumers use a simplified information search pattern, compared to high CRT consumers. To that end, in Experiment 2 we record eye movements while participants select one of two offers in 12 discount choice problems. Our objective is to test whether high cognitive reflective consumers are more likely, compared to low cognitive reflective consumers, to use an information search pattern which enable them to compute the final prices, and whether the probability of selecting the best deal depends on the application of this pattern.

**FIGURE 2:**
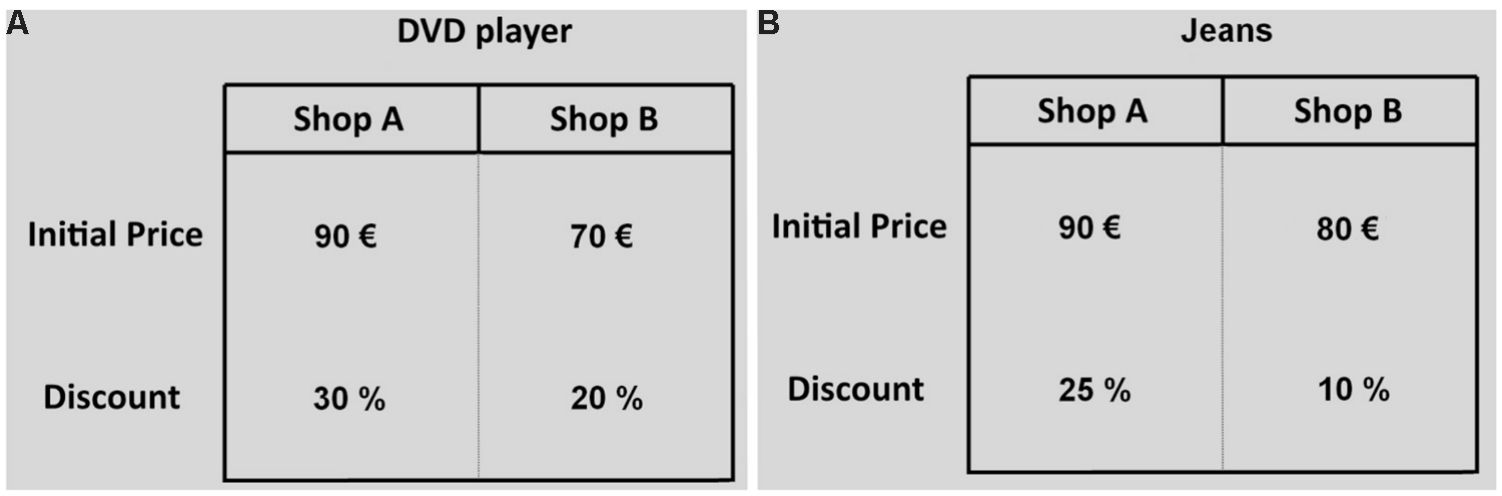
**Two examples of economic problems.** In the first example **(A)** the more worthwhile option is the one with the low initial price (Shop B), in the second example **(B)** the best option is the one with the high initial price (Shop A).

A correct procedure to compute the final prices requires the decision makers to compare the initial price of each option with the associated discount and engage themselves in a mental calculation. In terms of information search pattern, this procedure requires to use vertical eye movements (saccades between an initial price and the relative discount; see **Figure 2**) and long fixations. Vertical eye movements are expected because the initial price and the relative discount are displayed one above the other and this implies that the participants attention should move between these two pieces of information while calculating the final price. Long fixations are expected because the mental calculation of the final price is a complex cognitive operation that is composed of a set of distinct functional processes and longer fixations are commonly associated to deeper processing, such as deliberate consideration of information and planning (Velichkovsky, 1999; Velichkovsky et al., 2002; Eivazi and Bednarik, 2011; Glöckner and Herbold, 2011). On the other hand, people who use a partial approach in the selection of the commercial offer might exhibit a different information search pattern. For example, they might compare initial prices and discounts separately. In case that one of the alternatives yields a higher discount with a lower initial price, the decision makers choose that option. Alternatively, they may select the option with the higher discount or with the lower initial price. This simple comparison procedure involves horizontal eye movements (between option transitions) and do not require long fixations because it is less demanding in terms of cognitive effort.

### Method

Forty-four persons participated in the experiment, but nine of them were excluded (five due to poor calibration of the eye-tracking system and excessive number of trial losses, one because of the unusually high mean number of fixations across problems, and three because they reported, after the end of the experiment, that they were previously aware of some of the answers of the CRT). The mean age of the remaining 35 participants (14 males and 21 females) is 24.5 (SD = 3.47). The experiment was performed in accordance with the ethical standards laid down in the 1964 Declaration of Helsinki, and all participants gave informed consent prior to admission into the study.

After entering the lab, participants were informed of the nature of the “purchase decision ” task. Participants responded to 12 discount choice problems presented in a random order. For each problem the participants decided whether to buy a product (e.g., DVD player) at shop A or B. The two shops, A and B, sold the same identical product which was on sale with different initial prices and percentage discounts. For half of the problems the best deal is the one with the lowest initial price (see **Figure 2A**), for the other half it is the one with the highest initial price (**Figure 2B**). For each discount choice problem, participants had to select their response by pressing the corresponding key on the keyboard. Before starting the experiment, participants performed four practice trials. Choices and fixation times were automatically registered. After the discount choice experiment, subjects were asked to complete the Lipkus et al. (2001). Numeracy scale (Lipkus et al., 2001) and the CRT (Frederick, 2005)^4^.

#### Eye-Tracking Procedures

**FIGURE 3:**
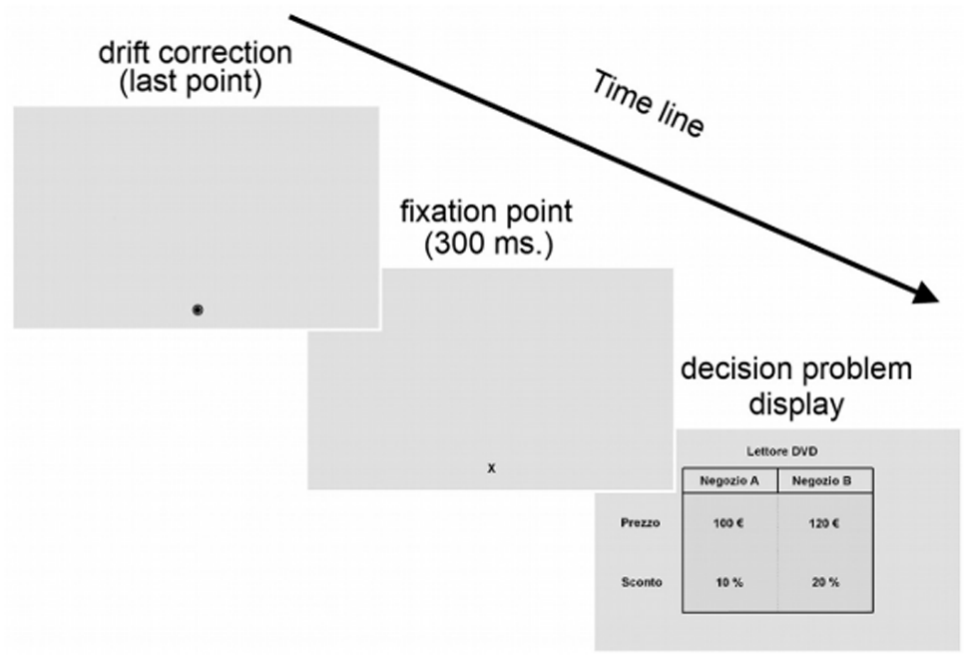
**Task and time course of a trial.** At the end of the drift correction, a fixation point was displayed for 300 ms on the bottom of the two possible options. Then, the economic problem was displayed onscreen until a response was made.

Participants were seated in a chair with a soft head restraint to ensure a viewing distance of 60 cm to the monitor. Presentation of the stimuli was performed using a custom made program written using the Matlab Psychophysical toolbox. Eye movements were monitored and recorded using an Eyelink II system (SR. Research Ontario Canada) with a sampling rate of 500 Hz. A fixation was defined as an interval in which gaze was focused within 1° of visual angle for at least 100 ms. (Manor and Gordon, 2003). A nine-point calibration was performed at the beginning of the experiment. Calibration phase was repeated until the difference between the positions of the points on the screen and the corresponding eye locations was less than 1°. After the calibration phase, a nine-point validation phase was performed (similarly to the calibration phase) to make sure that the calibration was accurate. Recalibrations were performed if needed, and eye-tracking was stopped if these were unsuccessful. Before the beginning of each trial a drift correction was performed. Then, a fixation point was presented in the same position as the last point of the drift correction for 300 ms. The fixation point was located at the bottom of the two possible options, outside the area covered by the picture representing the discount choice problem. Each discount choice problem was presented after the fixation point and remained on the screen until a response was made. Eye movements were recorded during the purchase decision problem display (**Figure 3**).

#### Eye-Tracking-Data

**FIGURE 4:**
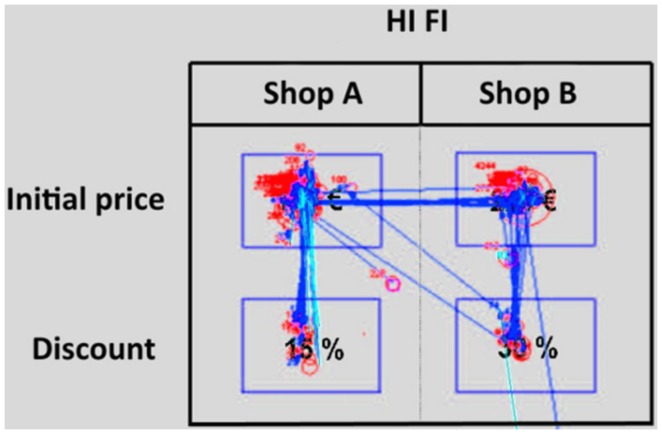
**Eye movement for one participant while responding to the discount choice problem.** Blue lines indicate saccades and red circles the location of fixations (the size of circle is proportional to the length of the fixations). The red number next to the red circle indicates length of the fixation. Blinks are indicated with light blue lines. The blue rectangles (not visible to the participants) represent the AOIs.

For each of the 12 problems, we defined four areas of interest (AOIs). All of them have a rectangular shape (**Figure 4**) and are centered on the four values (the two initial selling prices and the two discounts). The four AOIs include all the relevant information needed by the participants to make their decisions. Non-numerical information like the type of product (DVD player, microwave etc.) were not considered in the analysis because it was made clear at the beginning of the experiment that the two shops were delivering the same product and that the only difference between the two shops (A and B) were the initial selling prices and the discounts. None of the AOIs were adjacent to the others. This allowed us to avoid the possibility that small errors in the calibration procedures could result in an incorrect attribution of the eye movements parameters (fixations and saccades, see below). This is especially useful for those situations in which the parameter was located on the border of an AOI. AOIs do not cover the picture representing the discount choice problem entirely (but less than 8% of the screen) and never overlap. In this way, AOIs include only fixations and saccades whose interpretation is not ambiguous. Those parameters that were not located inside the AOIs were not considered in the analysis. However, although a large part of the picture is not included in any AOI, the majority of fixations observed fell inside the AOIs (88%). For each trial, we recorded the *average length of fixations* for each of the four AOIs, the *number of times* that the participant looked within an AOI, and the number and type of *saccades*, which correspond to the transitions made by the eye from one AOI to the next.

### Results

#### Choices: Numeracy and CRT

First, we investigated the influence of Numeracy on the participants’ ability to select the best offer. As in Experiment 1, Numeracy is measured by the number of correct answers given to the Lipkus scale and we treated Numeracy as a continuous variable. The binary dependent variable is the quality of commercial choice (i.e., the selection of the best deal). We decided to use a mixed regression model because the participants answered 12 discount choice problems which are considerably different from one another. They involve different products and different price ranges. Also, the absolute difference and the ratio of the initial and final prices change. This variability can be explained using the concept of “random factors,” i.e., variables whose levels are drawn “from a large (potentially very large) population in which the individuals differ in many ways, but we do not know exactly how or why they differ” (Crawley, 2007, p. 628). The mixed regression model (unlike ANOVA and simple linear regression) provides a way to take into account the added variation caused by differences between the levels of the random factors (Baayen et al., 2008; Fugard et al., 2011). As a consequence we included in our model a random factor called “Problem,” that discriminates among the 12 discount choice problems. Results from the logistic mixed model show the importance of Numeracy: higher Numeracy significantly increases the choice of the best options (β = 0.47, *Z* = 2.23, *p* = 0.03). In a second model, in addition to Numeracy, we added the factor CRT, considering the four levels of ability (from 0 to 3 correct answers). This model shows that Numeracy does not longer affect the choice of the best option (β = 0.26, *Z* = 1.18, *p* = 0.32), while CRT does (β = 0.82, *Z* = 3.42, *p* < 0.001). Given that these variables explore two related constructs we decided to verify the level of collinearity between them, so we calculated the variance inflation factor (VIF). Usually the largest VIF is taken to be a measure of the seriousness of collinearity among the predictors and the higher the value, the higher the collinearity. The definition of “high” is arbitrary but values higher than 4 or 5 are commonly considered an index of excessive correlation among explanatory variables. We found an index of 1.07 which allows us to exclude collinearity problems between the variables. As a second test, we also calculated the correlation between CRT and Numeracy to check for possible differences between our study and previous studies. We opted for the Spearman correlation coefficient, as it is not possible to assume neither that the variables are normally distributed nor that there is a linear correlation between them (both of which are necessary assumptions for the use of the Pearson correlation coefficient). Results show that the variables (CRT and Numeracy) are marginally correlated [Spearman correlation, *r*(35) = 0.29, *p* = 0.09]. In previous studies, Cokely and Kelley (2009) reported a significant correlation of 0.31. A stronger correlation was found in Finucane and Gullion (2010; *r* = 0.53) and in Liberali et al. (2012; *r* = 0.51 and *r* = 0.40). Despite the small size of our sample due to the constraints imposed by the eye-tracking device (participants have to be tested individually), the degree of correlation we found between Numeracy and CRT is in line with the correlation level obtained by Cokely and Kelley (2009).

**FIGURE 5:**
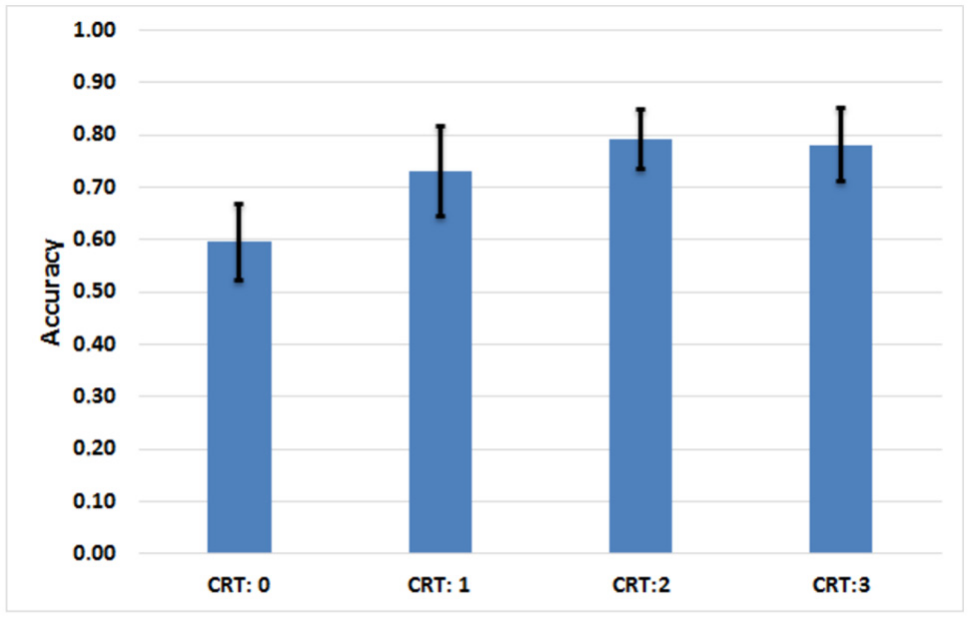
**Assessment of the relationship between the performance on the Cognitive Reflection Test (CRT) and the level of accuracy (proportion of best offers chosen) achieved by the participants in the 12 discount choice problems (mean and SD)**.

Results from the mixed model regression analyses are congruent with those obtained in Experiment 1, and they highlight the role of Cognitive Reflection in the selection of the best commercial offer. In **Figure 5** we report the relationship between performance on the CRT and the proportion of best offers chosen by the participants. **Table 3** details the summary statistics for the questionnaires and the eye-tracking analysis, while **Table 4** describe the parameters of the mixed models presented below.

**Table 3:** Descriptive statistics of the independent and dependent variables of Experiment 2.

	Average	SD
Cognitive Reflection Test (CRT) – number of correct responses	1.40	1.31
Numeracy – number of correct responses	8.11	1.94
Response time (ms)	20604	14586
Mean fix duration (ms)	323	83
Mean fix duration (shop A) (ms)	317	83
Mean fix duration (shop B) (ms)	314	75
Mean fix duration (prices) (ms)	366	111
Mean fix duration (discounts) (ms)	249	43
Total fixation time per decision (ms)	17061	14503
Total AOI fixation time per decision (ms)	16115	11702
Total fixation time per decision (shop A) (ms)	8874	6935
Total fixation time per decision (shop B) (ms)	7255	5589
Total fixation time per decision (prices) (ms)	12319	10110
Total fixation time per decision (discounts) (ms)	3804	2271
Number of total fixations per decision	49	35
Number of AOI fixations per decision	43	24
Number of AOI fixations per decision (shop A)	23	13
Number of AOI fixations per decision (shop B)	20	11
Number of between-option transitions per decision (prices)	4.90	1.94
Number of between-option transitions per decision (discounts)	1.99	0.99
Number of within-option transitions per decision (shop A)	7.15	4.10
Number of within-option transitions per decision (shop B)	6.41	3.93

*Eye-tracking statistics are calculated during the discount choice problem presentation.*

**Table 4:** A summary of the generalized linear mixed models used to test the relations among accuracy and questionnaires scores (Numeracy and CRT), among eye-movements parameters and CRT scores and among accuracy and eye-movements parameters.

Dependent variable	Predictors	Standardized coefficients	β	*Z/t*-value	*p*-value
**Choices**	Numeracy	0.47	*Z* = 2.23	=0.03
	**N° of obs. = 420**	**AIC = 494.4**	**BIC = 506.5**	**LogL = -244.2**
**Choices**	Numeracy	0.26	*Z* = 1.18	=0.32
	CRT	0.82	*Z* = 3.42	<0.001
	**N° of obs. = 420**	**AIC = 484.5**	**BIC = 500.7**	**LogL = -238.3**
**SI**	CRT	0.27	*t* = 5.68	<0.001
	**N° of obs. = 420**	**AIC = 120.4**	**BIC = 136.6**	**LogL = -56.6**
**LF-P**	CRT	0.33	*t* = 7.21	<0.001
	**N° of obs. = 420**	**AIC = 264.8**	**BIC = 281.0**	**LogL = -128.4**
**LF-D**	CRT	0.08	*t* = 1.57	=0.12
	**N° of obs. = 420**	**AIC = 90.5**	**BIC = 106.7**	**LogL = -41.3**
**NF-P**	CRT	0.20	*t* = 4.15	<0.001
	**N° of obs. = 420**	**AIC = 3805.3**	**BIC = 3821.5**	**LogL = -1898.7**
**NF-D**	CRT	0.06	*t* = 1.16	=0.25
	**N° of obs. = 420**	**AIC = 3115.7**	**BIC = 3131.9**	**LogL = -1553.8**
**SI**	LF-P	0.27	*t* = 5.55	<0.001
	NF-P	0.23	*t* = 5.04	<0.001
	**N° of obs. = 420**	**AIC = 65.7**	**BIC = 85.9**	**LogL = -27.8**
**Choices**	LF-P	0.49	*Z* = 1.77	=0.07
	NF-P	0.12	*Z* = 0.80	=0.42
	SI	**-**0.08	*Z* = **-**0.44	=0.66
	**N° of obs. = 420**	**AIC = 497.2**	**BIC = 517.4**	**LogL = -243.6**
**Choices**	LF-P	0.49	*Z* = 1.72	=0.08
	NF-P	0.14	*Z* = 0.71	=0.48
	**N° of obs. = 420**	**AIC = 495.4**	**BIC = 511.6**	**LogL = -243.7**
**Choices**	LF-P	0.53	Z = 2.27	=0.02
	**N° of obs. = 420**	**AIC = 493.9**	**BIC = 506.0**	**LogL = -244.0**

#### CRT and Eye Movements

As shown in the previous section, the effect of Numeracy scores on the proportion of best offers chosen is no longer significant when included in the same model with the CRT scores; therefore, in the following analyses, we focus only on the relationship between CRT and eye movements. Our research hypothesis predicts that participants with different levels of CRT use different decision processes and we investigate this prediction using three eye movement parameters (number and length of fixations and the Search Index (SI) calculated on the vertical/horizontal saccades).

##### Analysis of saccades

Vertical saccades are movements from one of the top cells (prices) to its corresponding lower cell (discount) or vice-versa (within-option or cue-based search). Horizontal saccades are movements from left to right (or vice-versa), so they connect the two prices or the two discounts (between-option or option-based search). In order to summarize the participants’ search patterns we calculated the (see Norman and Schulte-Mecklenbeck, 2010 for a theoretical description and Glöckner and Betsch, 2008 for an application of the SI to an eye-tracking study). The SI is calculated by subtracting the number of between-option transitions from the number of within-option transitions and dividing this difference by the sum of both transitions. SI ranges from -1 to +1 and negative values indicate more between-option searches, while positive values indicate more within-option searches. Within-option transitions are necessary to link the initial price with the corresponding discount, in order to calculate the final price. Conversely, between options transitions in our purchase decision task do not give rise to any economically relevant comparison.

We run a linear mixed model to test the hypothesis that participants who obtained high scores in the CRT were more prone to use within-option transitions. Results indicate that higher CRT scores significantly increase the proportion of vertical (within-option) transitions (higher SI, β = 0.27, *t* = 5.68, *p* < 0.001).

##### Analysis of the average length of fixations

The data were treated in the following way: we calculated the average length of fixations for each AOI and used the natural logarithm transformation of the values in order to approximate a normal distribution (Newell and Rosenbloom, 1981). The average length of fixations for the two price areas are highly correlated, [*r*(35) = 0.94, *p* < 0.001], like the average length of fixations for the two discount areas [*r*(35) = 0.70, *p* < 0.001]. Conversely, the correlations between the average length of fixations for the each of the two prices with each of the two discounts are not significant. Given these results, we considered the areas of the two prices as a single area of interest and, similarly, we treated the two discounts as a single area of interest. The factors length of fixations for the Price Area (LF-P) and length of fixations for the Discount Area (LF-D) indicate the average length of fixations on the two macro-areas.

We run a linear mixed model to evaluate the hypothesis that the score obtained in the CRT is positively correlated with LF-P. Results indicate that a higher score in the CRT significantly increases the LF-P (β = 0.33, *t* = 7.21, *p* < 0.001). The same analysis was repeated to evaluate the association between CRT and LF-D, but this time the predictor was not statistically significant (β = 0.08, *t* = 1.57, *p* = 0.12).

##### Fixation counts

We recorded how many times the participants looked at a certain AOI and, paralleling the analyses on the average fixation length, we aggregated the AOIs into two macro-areas: the Price Area and the Discount Area. The factors number of fixations in the Price (NF-P) and Discount Areas (NF-D) indicate the average number of fixations within each of the two macro-areas. Then, we ran two linear mixed models and tested whether the score obtained in the CRT is positively correlated with the NF-P and NF-D factors. Similarly to what we observed in the analysis of the average length of fixations, we found that participants who have a higher score in the CRT have a higher number of fixations in the Price Area (β = 0.20, *t* = 4.15, *p* < 0.001) but not in the Discount Area (β = 0.06, *t* = 1.16, *p* = 0.25).

Overall, results of the saccades and fixation analyses show that participants with different CRT scores used different information search patterns. In particular, the performance on the CRT is positively related to the proportion of within-option transitions and the average length and number of fixations in the Price Area.

##### Relationship between the depth of the elaboration process and the within-option search strategy

We studied the relationship between fixations (average length and number of fixations) and saccades (SI) because we expected to find an association between the depth of the elaboration process and the within-option search strategy. For the SI a linear mixed model with the factors LF-P and NF-P shows that the average length and number of fixations is significantly associated with the proportion of within-option transitions. More precisely, the proportion of within-option transitions increases when the length (β = 0.27, *t* = 5.55, *p* < 0.001) and the number of fixations (β = 0.23, *t* = 5.04, *p* < 0.001) increase within the Price Area. This result indicates that the participants who processed the problem information more deeply used a within-option evaluation strategy.

#### Eye Movements and Choice Accuracy

Finally, we test the relationship between the information search patterns and the probability of selecting the best offer. We run a series of logistic mixed model that includes the eye movement parameters that had a significant effect in the previous analyses (SI, the LF-P, and the NF-P) using the backward elimination procedure and we test whether they are good predictors of choice accuracy (i.e., how often the participants chose the more advantageous option). The initial model (AIC = 497.2) which includes the three predictors shows that LF-P is marginally significant (β = 0.49, *Z* = 1.77, *p* = 0.07), while NF-P (β = 0.12, *Z* = 0.80, *p* = 0.426), and SI (β = -0.08, *Z* = -0.44, *p* = 0.663) are not. We eliminated SI, the least relevant predictor, and we run a model with the variables LF-P and NF-P. The fit of the model improves (AIC = 495.4), LF-P has a marginally significant effect (β = 0.49, *Z* = 1.72, *p* = 0.08), while NF-P does not (β = 0.14, *Z* = 0.71, *p* = 0.48). Finally, we eliminated the variable NF-P and now the model shows another improvement in the goodness-of-fit (AIC = 493.9) and a fully significant effect of LF-P (β = 0.53, *Z* = 2.27, *p* = 0.023).

#### Information Search Patterns for High and Low CRT Scores

**FIGURE 6:**
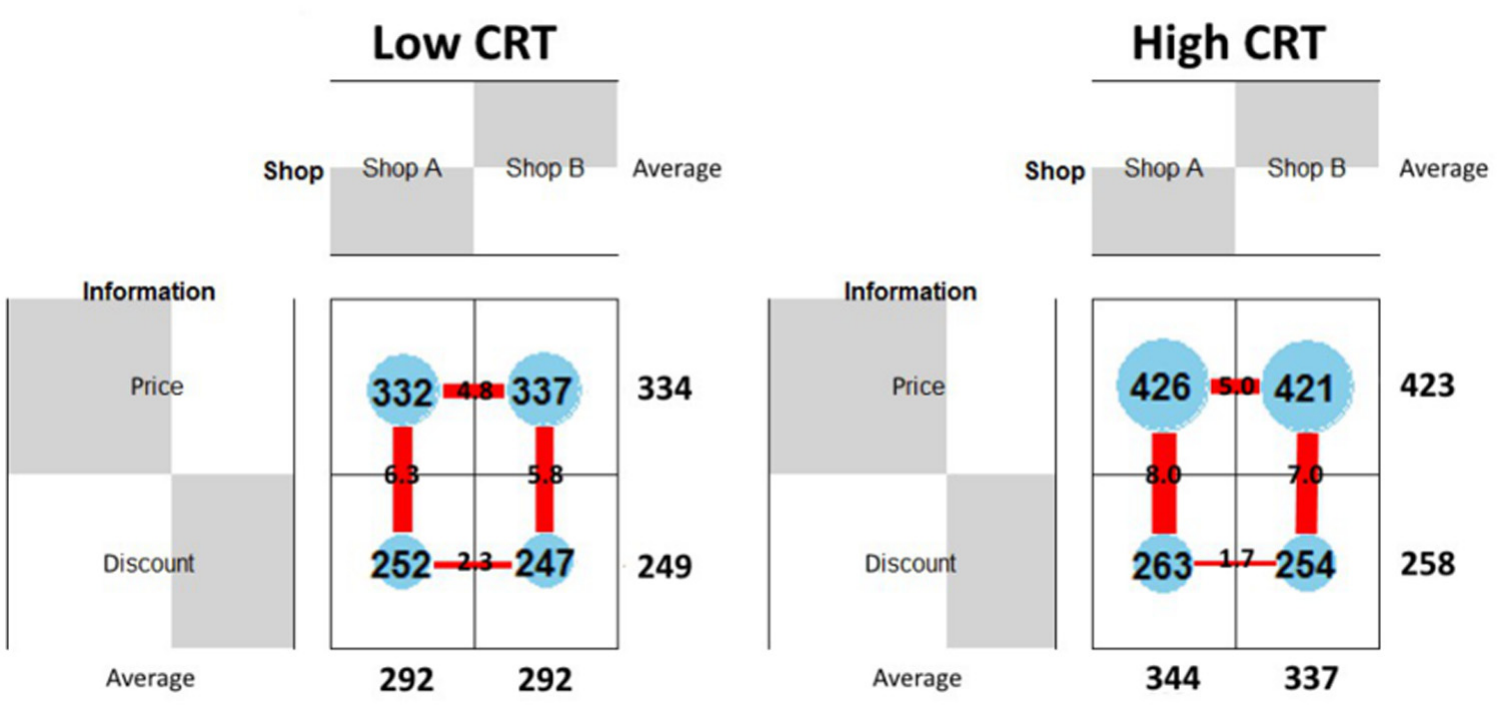
Information processing; for low **(left)** and high **(right)** CRT participants. The numbers within the blue circles indicates the average length (in milliseconds) of fixations for the four AOIs (the radius of the circles is proportional to the average length of fixations). The numbers superimposed on the red lines indicates the average number of saccades for the four types of transitions the thickness of the lines is proportional to the average number of saccades).

In order to give a general picture of the information search patterns used by participants with different CRT scores we divided them into two groups – high and low CRT – and we compared their search patterns. Participants who gave two or three correct answers were classified as “high CRT” (17 participants) and those who gave one or zero correct answers as “low CRT” (18 participants). As shown in **Figure 6** the average length of fixations on the price areas for high CRT participants is almost 100 ms longer compared to low CRT participants (low CRT = 334 ms; high CRT = 423 ms). Conversely no differences were found on the Discount areas (low CRT = 249 ms; high CRT = 258 ms). These results suggest that high CRT participants are more likely to engage themselves in the calculation of the final prices and that the mental calculation of the final discounted prices was performed while fixating on the price areas, which greatly simplified the maintenance of the initial prices in the working memory. The two groups differ also in the number of vertical saccades (low CRT = 6.05; high CRT = 7.5) but not in the number of horizontal saccades (low CRT = 3.55; high CRT = 3.35), which is in accordance with the fact that only saccades between price and discount values are useful for the mental calculation.

In general, we suggest that high CRT participants are more likely to remain focused on the initial prices, using vertical saccades to read and maintain the discount values in working memory. This information search pattern is suitable for the mental calculation of the final price. Conversely, low CRT participants are more likely to use a less specific information search pattern which does not include the mental calculation of the final price (see **Figure 6** below).

### Conclusion

Reported findings suggest that the ability to choose the best option in a simple purchase decision problem depends not only on numerical skills, but also on cognitive impulsivity as measured by the CRT. We also found that the CRT predicts the information search pattern used which, in turn, affects the quality of purchase decision. Specifically, we found that consumers with low levels of Cognitive Reflection do not use a sufficiently detailed information search pattern and pick a low proportion of the more advantageous offers. Experiment 2 extends the findings of Experiment 1 to a series of choices about a variety of consumers goods, giving a more robust description of consumers’ behavior.

## General Discussion

Consumers must often decide which is the most advantageous economic option among a list of alternatives, and the literature shows that sometimes they choose poorly in conditions of risk or uncertainty (Starmer, 2000; Kahneman, 2003; Bonini et al., 2004; Shu, 2006). Consumers also show poor performance when coping with riskless choice. For example, when they have to find which is the cheapest goods bundle for a certain product or – as in the present study – which is the best deal (see Armstrong, 2010 for a comprehensive analysis on this point).

Our research aims to expand the current knowledge about the characteristics of economic behavior by studying the influence of two cognitive skills: Numeracy and Cognitive Reflection. While it has already been shown that higher levels of cognitive skills (e.g., ability to plan, memory, etc.) are associated with better choices (Burks et al., 2009; Christelis et al., 2010), the comprehension of the link between decision processes and cognitive skills is still limited. In addition, the association between CRT scores and choices has been little studied in the context of the economic domain – one of the few examples is the study by Finucane and Gullion (2010).

In a series of purchase decision problems, our experimental findings indicate that consumers with high CRT scores and high Numeracy are significantly more likely to make the more advantageous choice, compared to people who are more cognitively impulsive and/or less numerate. Starting from our experimental hypotheses this result can be explained by the different decision processes that characterize participants with high/low scores in the CRT and Numeracy Test. In Experiment 1 we show that consumers with low Numeracy use a partial decision approach, i.e., they use only part of the numeric information provided and they do not compute all the operations needed to identify the best option. Hence, they select the worst option much more frequently, compared to highly numerate consumers. In Experiment 2 we found that both CRT and Numeracy have an influence, with a particularly strong effect of the former variable, on the aptness of the purchase decisions. It is relevant to note that previous studies (see for example Del Missier et al., 2012) indicate the CRT scores are partly influenced by Numeracy. In the present study we did not estimate the relative importance of the two sides of CRT (reflexiveness and Numeracy) but the results from Experiment 2 and the findings of Del Missier et al. (2012) suggest that the quality of purchase choices depend on the (in) capacity of inhibiting an intuitive (although) wrong answer and on Numeracy. In addition to the effect of the cognitive skills on choice, the analyses on the eye movement data show that people with lower CRT scores adopt a shallow search pattern, which is associated with worse performance in the purchase decision task.

On the whole, both experiments support our experimental hypotheses that people with greater cognitive skills are more likely to use thorough decision-making processes, which produce a higher number of optimal choices.

We believe that the reported findings can contribute to the growing literature on the vulnerability of consumers (see OECD, 2010 consumer policy toolkit). As shown by a recent survey on proficiency in key information-processing skills among working-age adults (OECD, 2013), insufficient Numeracy is a widespread phenomenon: only one adult out of three is able to act upon and correctly interpret relatively simple data and statistics in texts, tables and graphs. The great extent of the phenomenon indicates that Numeracy is certainly one factor in consumer vulnerability, as shown also in our study. This vulnerability appears also in very common economic problems, where consumers make decisions based upon the computation of simple numeric information such as integers or percentages (see Yin and Dubinsky, 2004 on the influence of comparative price formats and Graffeo and Bonini, 2015, on the relationship between Numeracy and comparative price formats). However, reported findings add another factor to the vulnerability list: lack of cognitive reflection or cognitive impulsivity. Consumers with low CRT scores tend to choose poorly, most likely because their first impression of “the best deal” is not corrected by further information processing (e.g., the computation of the final prices).

These findings are of interest for both marketing experts and consumer policy specialists since there is a recent and growing concern in the European Union about unfair commercial practices, and consumer rights in general (European Parliament, and Council, 2005, 2011). Furthermore, findings suggest that the attributes that characterize the effective decision-maker include particular cognitive skills other than financial or mathematical knowledge. This conclusion is congruent with the broad definition of “financial capability” provided by OECD (PISA, 2012, p. 13) where it is stated that: “Additionally [to knowledge and understanding of financial concepts and risks], financial literacy involves skill in managing the emotional and psychological factors that influence financial decision-making.” We believe that the ability to counteract the first (and false) impression of goodness in a commercial setting is one of these crucial psychological factors.

Finally, our findings show that the use of some specific decision processes is an indicator that certain factors of vulnerability are at work; a more detailed knowledge of these processes could facilitate the development of comprehensive guidelines that illustrate how to prevent some recurrent mistakes. In this way it would be possible to mitigate the influence of the vulnerability factors, in particular within socially relevant domains.

## Conflict of Interest Statement

The authors declare that the research was conducted in the absence of any commercial or financial relationships that could be construed as a potential conflict of interest.

## Footnotes

1 For example, let’s consider the Microwave oven scenario, where the oven is sold at Shop A with an initial price of 70 with a 20% discount and at Shop B with an initial price of 90 with a 30% discount. Some participants could consider negligible the difference between 70€ and 90€; in that case they could ignore the information “initial price” and base their decision only on the remaining characteristic, the percentage discount (for a description of a similar process see Leland, 1994; Birnbaum and Gutierrez, 2007). On the contrary, other participants could consider that the 20€ difference is important enough to be considered, so their choice is based on all the provided information.

2 We run the mediation model presented below with the addition of the factor “product.” The relationships between variables are not affected at all by the factor “product.” The factors Numeracy and Decision Approach remain significant, while the factor “product” is never significant. Here we present the relationships between the variables using a series of correlation analyses. Correlation between Numeracy and Choice: DVD Player [*r*(50) = 0.210; *p* = 0.144]. Microwave oven [*r*(51) = 0.158; *p* = 0.267]. Mobile phone [*r*(52) = 0.176; *p* = 0.213]. Correlation between Decision approach and Choice: DVD Player [*r*(50) = 0.234; *p* = 0.102]. Microwave oven [*r*(51) = 0.032; *p* = 0.821]. Mobile phone [*r*(52) = 0.272; *p* = 0.051]. Correlation between Numeracy and Decision approach: DVD Player [*r*(50) = 0.231; *p* = 0.107]. Microwave oven [*r*(51) = 0.262; *p* = 0.063]. Mobile phone [*r*(52) = 0.136; *p* = 0.335].

3 In both experiments we report standardized beta coefficients, calculated following the procedure proposed by Goodman (1972) and discussed by Menard (2004).

4 The original studies about the Lipkus Numeracy scale (Lipkus et al., 2001) and the CRT (Frederick, 2005) indicate that the two scales have different difficulty levels (68 and 41% of correct answers, respectively). This difference is relevant in a context where the decision problem is difficult, because a more difficult scale may differentiate people more effectively (i.e., differentiate them at the appropriate level of performance) that a less difficult scale.
